# Antimicrobial Resistance Knowledge, Training Exposure, and Prescribing Influences Among Community Health Workers in Kaduna State, Nigeria

**DOI:** 10.7759/cureus.111045

**Published:** 2026-06-17

**Authors:** Muhammad T Adamu, Drusilla B Adams, Maryam C Adamu, Idris Adamu, Henry Okafor

**Affiliations:** 1 Department of Internal Medicine, Modibbo Adama University Teaching Hospital, Yola, NGA; 2 Department of Pharmacy, Kaduna State Ministry of Health, Kaduna, NGA; 3 Department of Pharmaceutical Services, Federal Teaching Hospital, Gombe, NGA; 4 Department of Public Health, Ahmadu Bello University, Zaria, NGA; 5 Department of Community Medicine, Nnamdi Azikiwe University Teaching Hospital, Nnewi, NGA

**Keywords:** antimicrobial resistance, antimicrobial stewardship, community health workers, nigeria, prescribing behavior

## Abstract

Introduction: Antimicrobial resistance (AMR) is a major public health threat, particularly in low- and middle-income countries where inappropriate antibiotic use remains common. Community Health Workers (CHWs) are important providers of primary healthcare (PHC) in Nigeria, yet evidence on their AMR knowledge, stewardship training, and prescribing influences remains limited. This study assessed AMR/AMS knowledge, training exposure, prescribing attitudes, and factors influencing antimicrobial prescribing among CHWs in Kaduna State, Nigeria.

Methods: A descriptive cross-sectional study was conducted among 286 CHWs selected from 27 government-owned PHC facilities in Kaduna State using multistage sampling. Data were collected between August and September 2025 using a structured self-administered questionnaire. Data were analyzed using IBM Statistical Package for the Social Sciences version 25.0 (IBM Corp., Armonk, NY). Descriptive statistics were presented as frequencies and percentages, while chi-square tests assessed associations between AMR/AMS training, knowledge level, and attitude level.

Results: Most respondents had heard of AMR (87.4%), correctly identified bacteria as the target of antibiotics (90.9%), and recognized that inappropriate antibiotic use contributes to resistance (92.7%). Only 20.6% had received formal AMR/AMS training. Although many respondents reported guideline use (68.6%) and prescription documentation (70.3%), 62.3% prescribed antibiotics based on experience without diagnostic tools, while 38.1% reported patient demand-influenced prescribing decisions. Formal AMR/AMS training was significantly associated with both knowledge level and attitude level.

Conclusion: Despite generally good AMR knowledge, formal stewardship training among CHWs was limited. Strengthening AMR/AMS training, diagnostic support, supervision, and guideline-based prescribing may improve antimicrobial stewardship at the PHC level.

## Introduction

Antimicrobial resistance (AMR) has emerged as a major public health threat. It places millions at risk of difficult-to-treat infections by undermining the effectiveness of antibiotics [1-4]. Between 20% and 50% of all antimicrobial use is deemed inappropriate according to global estimates, especially in settings where infectious diseases account for substantial morbidity and mortality [5]. AMR accounted for approximately 1.27 million deaths in 2019, with more than five million deaths associated with resistant infections globally. The highest burden was recorded in sub-Saharan Africa [6].

Low- and middle-income countries (LMICs) are particularly vulnerable as weak regulatory systems, limited diagnostic capacity, inadequate surveillance, and gaps in access to trained healthcare providers perpetuate inappropriate antimicrobial use [7-10]. At the Primary Healthcare (PHC) level, antibiotic prescription rates as high as 98.2% were reported in Northern Nigeria [11]. This highlights the need to strengthen antimicrobial stewardship (AMS) beyond hospitals.

PHC remains a central pillar to achieving universal health coverage, especially in underserved communities [7,12]. In Nigeria, task-shifting has positioned community health workers (CHWs) as essential providers of care [8-10,13]. This positions them on the front lines of antimicrobial use with limited supervision, diagnostic support, treatment guidelines, and professional development [12,14-16]. CHWs may be required to prescribe under considerable uncertainty.

Knowledge of AMR may not translate into stewardship-aligned practice. This is especially true in LMICs, where social and institutional factors may influence prescribing behaviors [14-17]. Providers may resort to empirical prescribing or prescribing under patient pressure even when not clearly indicated [12,15,16].

In Nigeria, studies have examined antimicrobial prescribing patterns and AMR awareness among healthcare workers, but fewer have focused specifically on AMR/AMS knowledge, exposure to formal training, prescribing-related attitudes, and contextual prescribing influences among CHWs at the PHC level [11,18]. Understanding these factors is important because CHWs often provide first-contact care in settings where diagnostic support, treatment guidelines, and supervision may be limited [10,12,16].

Evidence from Northern Nigeria indicates that antibiotic prescription rates at the PHC level reach 98.2%, with the majority of these prescriptions driven by empirical decision-making in the absence of diagnostic confirmation. This high prevalence of empirical prescribing highlights an urgent need for targeted investigations into the frontline workforce to inform context-specific stewardship interventions [11].

The primary objective of this study was to assess the level of AMR/AMS knowledge, the extent of formal training exposure, and attitudes toward antimicrobial prescribing among CHWs in Kaduna State, Nigeria. It also aimed to identify the contextual factors influencing their antimicrobial prescribing decisions, with the goal of generating evidence that can inform context-specific stewardship interventions aimed at strengthening antimicrobial use at the PHC level.

## Materials and methods

Study design and setting

This was a descriptive cross-sectional study conducted among CHWs in Kaduna State, Northwestern Nigeria. Kaduna State comprises 23 local government areas (LGAs) distributed across three senatorial districts: Northern, Central, and Southern. The study was conducted at selected government-owned PHC facilities across the state.

Study population

The study population comprised registered CHWs actively practicing in government-owned PHC facilities in Kaduna State during the study period. These included Community Health Officers (CHOs), Community Health Extension Workers (CHEWs), Junior Community Health Extension Workers (JCHEWs), and Community Nurses involved in PHC antimicrobial prescribing.

These cadres are distinguished by their training and certification. JCHEWs possess a certificate in community health following a two-year training program, while CHEWs hold a diploma after a three-year training program. CHOs hold a Higher National Diploma after an additional year of specialized training. Community nurses undergo a two-year training program and are certified by the Nursing and Midwifery Council of Nigeria [19,20].

Eligible participants were CHWs currently practicing at the selected PHC facilities who had worked there for at least six months and who provided informed consent to participate. Respondents were excluded if they were on leave during data collection. Trainees who were not independently involved in prescribing and those who declined participation were also excluded. Other healthcare cadres outside the prespecified CHW categories were excluded from the study.

Sample size determination

The estimated number of CHWs in Kaduna State, provided by the Kaduna State Ministry of Health for 2025, was approximately 1,000. This estimate was used to calculate the study's sample size. The minimum sample size was calculated using Cochran’s formula for a single proportion:

$n = \frac{Z^2 pq}{d^2}$

where n is the initial sample size, Z is the standard normal deviate for a 95% confidence level (1.96), p is the estimated proportion, q = 1 − p, and d is the margin of error (0.05).

A prevalence of 50% was used because no prior CHW-specific prevalence estimate was available for the primary outcome in Kaduna State, which yields the most conservative sample size estimate. This gave an initial sample size of 384. Because the target population was less than 10,000, a finite population correction was applied using:

$n_f = \frac{n}{1 + \frac{n - 1}{N}}$

where n_f_ is the adjusted sample size, n is the initial sample size, and N is the estimated study population. This produced a minimum sample size of 278.

Sampling technique

A multistage sampling technique was used to ensure adequate geographic coverage across Kaduna State. First, three LGAs were selected from each of the three senatorial districts using simple random sampling, yielding a total of nine LGAs. The selected LGAs were Kudan, Sabon Gari, and Zaria from the Northern district; Chikun, Igabi, and Birnin Gwari from the Central district; and Jema’a, Kachia, and Zangon Kataf from the Southern district.

Second, three government-owned PHC facilities were randomly selected from each chosen LGA, giving a total of 27 PHC facilities. Third, eligible CHWs within these selected facilities were listed. Respondents were selected through simple random sampling, proportional to staff strength at each facility.

Data collection instrument

Data were collected using a structured, self-administered questionnaire developed after reviewing relevant literature (see the Appendix). The questionnaire included sections on sociodemographic characteristics, AMR/AMS awareness and knowledge, formal AMR/AMS training, prescribing-practice indicators, attitudes toward prescribing, and perceived factors influencing antimicrobial use.

To enhance content validity, the instrument underwent expert review by clinicians and public health specialists experienced in AMS. The experts assessed the relevance, clarity, and contextual appropriateness of the items for use among CHWs in the Nigerian PHC setting. The questionnaire was subsequently pretested among 20 CHWs outside the study area to assess comprehensibility and completion time. Feedback from the pretest led to minor revisions to wording and structure. Data from the pretest were not included in the final analysis.

Data collection procedure

Data were collected between August 11, 2025, and September 10, 2025, by trained research assistants. The questionnaires were administered during scheduled visits to the selected PHC facilities. Participants completed the questionnaires anonymously after receiving an explanation of the study objectives and providing consent. Completed questionnaires were checked for completeness before collation.

Study variables

The primary variables were AMR/AMS knowledge level, formal AMR/AMS training exposure, stewardship-related prescribing practices, and attitude toward antimicrobial prescribing. Knowledge and attitude levels were classified as poor, fair, or good using predefined investigator-developed scoring criteria established prior to data analysis to facilitate interpretation of questionnaire responses. Knowledge of antimicrobial use and stewardship was assessed using five structured questions. Each correct response was assigned one point, while incorrect or uncertain responses were scored zero, yielding a total score ranging from 0 to 5. Knowledge scores were categorized as poor (0-1), fair (2-3), and good (4-5). Attitude toward AMS was assessed using six Likert-scale statements scored on a five-point scale ranging from strongly agree to strongly disagree. Negatively worded statements were reverse-coded such that higher scores reflected more stewardship-friendly attitudes. Total attitude scores ranged from 6 to 30 and were categorized as poor (6-14), fair (15-22), and good (23-30). These scoring categories were developed specifically for this study and were not derived from a previously validated scoring instrument.

Data management and statistical analysis

Completed questionnaires were reviewed for completeness, coded, and entered into a secure electronic database. The dataset was cleaned and checked for consistency before analysis. Statistical analysis was performed using the IBM Statistical Package for the Social Sciences, version 25.0 (IBM Corp., Armonk, NY).

Descriptive statistics were used to summarize respondents’ characteristics, knowledge, training exposure, attitudes, and prescribing practices. Chi-square tests were used to assess associations between knowledge level, AMR/AMS training, attitude level, and selected sociodemographic or prescribing-related variables. Statistical significance was set at p < 0.05.

Ethical considerations

The study was conducted in accordance with the Declaration of Helsinki. Ethical approval was obtained from the Health Research and Ethics Committee of the Kaduna State Ministry of Health (NHREC/17/03/2018). Administrative clearance was also obtained from the Kaduna State Primary Health Care Board before the commencement of data collection.

All participants were informed of the study’s purpose, and informed consent was obtained prior to participation. No personally identifiable information was collected. Participation was voluntary, and respondents were informed of their right to withdraw from the study at any stage without penalty.

## Results

A total of 286 eligible respondents participated in the study, exceeding the minimum sample size. The largest proportion was within the 26-35 age group (n = 105, 36.7%), with an average age of 37.44 ± 10.88 years. Gender distribution was nearly balanced, with 149 (52.1%) male and 137 (47.9%) female participants. CHOs were the largest cadre (n = 109, 38.1%), followed by CHEWs (n = 100, 35.0%), JCHEWs (n = 43, 15.0%), and Community nurses (n = 34, 11.9%). Most respondents (n = 116, 40.6%) reported having one to five years of professional experience, while 74 respondents (25.9%) had over a decade of experience. The sociodemographic and professional characteristics of the respondents are summarized in Table 1.

**Table 1:** Sociodemographic and professional characteristics of respondents JCHEW: Junior Community Health Extension Worker; CHEW: Community Health Extension Worker; CHO: Community Health Officer

Variable	Frequency	Percentage (%)
Age		
18-25	40	14.0
26-35	105	36.7
36-45	69	24.1
46-55	49	17.2
56 and above	23	8.0
Mean (standard deviation)	37.44 (10.88)	
Sex		
Male	149	52.1
Female	137	47.9
Marital status		
Single	116	40.6
Married	137	47.9
Divorced	15	5.2
Widowed	18	6.3
Cadre		
JCHEW	43	15.0
CHEW	100	35.0
CHO	109	38.1
Community nurses	34	11.9
Years of experience		
<1 year	23	8.0
1-5 years	116	40.6
6-10 years	73	25.5
>10 years	74	25.9

Most respondents had heard of AMR (n = 250, 87.4%), correctly identified bacteria as the appropriate target of antibiotics (n = 260, 90.9%) and recognized that inappropriate antibiotic use can cause resistance (n = 265, 92.7%). Awareness of AMS was lower: 200 respondents (69.9%) had heard of it, and 218 (76.2%) correctly understood it to mean ensuring appropriate antibiotic use. Only 59 respondents (20.6%) had ever received formal AMR/AMS training, while 227 (79.4%) had not. The respondents' AMR knowledge, AMS awareness, and training exposure are summarized in Table 2.

**Table 2:** AMR/AMS awareness, knowledge, and training exposure AMR: antimicrobial resistance; AMS: antimicrobial stewardship

Variable	Frequency	Percentage (%)
Have you heard of the term “Antimicrobial resistance”?		
Yes	250	87.4
No	36	12.6
Antibiotics are effective against which of the following		
Bacteria	260	90.9
Viruses	1	0.3
Both	23	8.0
Don’t know	2	0.7
Can inappropriately use of antibiotics cause resistance?		
Yes	265	92.7
No	4	1.4
Not sure	17	5.9
Completing a full course of antibiotics reduces the risk of AMR		
Yes	265	92.7
No	21	7.3
Have you heard of antimicrobial stewardship?		
Yes	200	69.9
No	86	30.1
Antimicrobial stewardship means		
Preventing the sale of antibiotics	8	2.8
Ensuring the appropriate use of antibiotics	218	76.2
Recommending herbal alternatives	2	0.7
Not sure	58	20.3
Have you ever received formal training on AMR/AMS?		
Yes	59	20.6
No	227	79.4

Overall, AMR knowledge was categorized as poor, fair, or good based on respondents' total knowledge scores. Most respondents had good knowledge (n = 218, 76.3%), while 63 respondents (22.0%) had fair knowledge and five respondents (1.7%) had poor knowledge.

Most respondents agreed or strongly agreed that they followed standard treatment guidelines when prescribing antimicrobials (n = 196, 68.6%) and documented antimicrobial prescriptions (n = 201, 70.3%). However, 178 respondents (62.3%) also agreed or strongly agreed that they prescribed antibiotics based on experience even without diagnostic tools, while 109 respondents (38.1%) agreed or strongly agreed that patient demand influenced their prescribing decisions. In addition, 77 respondents (26.9%) agreed or strongly agreed that they prescribed antibiotics for common cold symptoms, and 60 respondents (21.0%) agreed that they prescribed antibiotics for malaria. It was also observed that 43.4% (n=124) and 35.3% (n = 101), respectively, remained neutral when asked about antimicrobial prescriptions for cold and malaria. The distribution of prescribing-related responses is summarized in Table 3.

**Table 3:** Attitudes toward antimicrobial prescribing among respondents

S/N	Statements	Strongly agree, n (%)	Agree, n (%)	Neutral, n (%)	Disagree, n (%)	Strongly disagree, n (%)
1	I prescribe antibiotics based on my experience, even without diagnostic tools	17 (5.9)	161 (56.4)	53 (18.5)	43 (15.0)	12 (4.2)
2	Patient demand influences my decision to prescribe antibiotics	4 (1.4)	105 (36.7)	64 (22.4)	95 (33.2)	18 (6.3)
3	I prescribe antibiotics for common cold symptoms	5 (1.7)	72 (25.2)	124 (43.4)	66 (23.1)	19 (6.6)
4	I prescribe antibiotics for malaria	8 (2.8)	52 (18.2)	101 (35.3)	94 (32.9)	31 (10.8)
5	I follow a standard treatment guideline when prescribing antimicrobials	76 (26.6)	120 (42.0)	76 (26.6)	13 (4.5)	1 (0.3)
6	I document all antimicrobial prescriptions	58 (20.3)	143 (50.0)	70 (24.5)	13 (4.5)	2 (0.7)

Factors influencing antibiotic prescribing decisions are presented in Table 4. Laboratory results were the most frequently reported influence on prescribing decisions (n = 275, 96.2%), followed by patient symptoms (n = 238, 83.2%) and previous patient response to similar symptoms (n = 76, 26.6%). Other reported influences included stock availability in the facility (n = 51, 17.8%), pressure from patients or relatives (n = 39, 13.6%), lack of diagnostic tools (n = 31, 10.8%), lack of guidelines (n = 15, 5.2%), and lack of supervision or oversight (n = 7, 2.4%). Respondents could select more than one option; therefore, percentages may exceed 100% when summed.

**Table 4:** Factors influencing antimicrobial prescribing decisions among respondents

Variable	Frequency (n)	Percentage (%)
What factors influence your decision to prescribe antibiotics?		
Patient symptoms	238	83.2
Laboratory results	275	96.2
Pressure from patients and relatives	39	13.6
Stock availability in the facility	51	17.8
Previous patient response to similar symptoms	76	26.6
Lack of supervision or oversight	7	2.4
Lack of diagnostic tools	31	10.8
Lack of guidelines	15	5.2
How often do you receive supervision on prescribing practices?		
Never	61	21.3
Rarely	131	45.8
Occasionally	59	20.6
Regularly	30	10.5
Always	5	1.7

Formal AMR/AMS training was significantly associated with both AMR knowledge level and attitude level. All respondents (n = 59, 100.0%) who had received formal AMR/AMS training had good knowledge, compared with 159 respondents (70.0%) without formal training. Similarly, respondents who had received formal AMR/AMS training had a higher proportion of respondents with a good attitude than those without formal training. The associations of formal AMR/AMS training with knowledge and attitude levels are shown in Table 5.

**Table 5:** Association between formal AMR/AMS training with knowledge and attitude levels among respondents AMR: antimicrobial resistance; AMS: antimicrobial stewardship

Outcome variable	Formal AMR/AMS training	Poor, n (%)	Fair, n (%)	Good, n (%)	X^2 ^value	p value
Knowledge level	Yes	0 (0.0)	0 (0.0)	59 (100.0)	23.187	<0.001
	No	5 (2.2)	63 (27.8)	159 (70.0)		
Attitude level	Yes	2 (3.4)	29 (49.2)	28 (47.5)	21.595	<0.001
	No	7 (3.1)	178 (78.4)	42 (18.5)		

## Discussion

AMR remains a major public health concern, particularly in low- and middle-income countries where inappropriate antibiotic use is common. In this study, most respondents demonstrated high awareness and good knowledge of AMR and AMS, but only a minority had received formal AMR/AMS training. This pattern is consistent with evidence from the WHO African Region, which shows increasing AMR awareness but uneven access to structured stewardship education among frontline health workers [4,18].

Formal AMR/AMS training was significantly associated with both knowledge and attitude. All trained respondents had good knowledge, and a higher proportion of trained respondents had favorable attitudes than those without training. This suggests that structured training may play an important role in strengthening stewardship-related knowledge and attitudes among CHWs. Unlike general awareness, formal training may provide practical guidance on guideline use, diagnostic reasoning, and appropriate antimicrobial decision-making.

Attitudinal responses showed that antimicrobial prescribing may be shaped by factors beyond knowledge alone. A substantial proportion of respondents reported prescribing based on experience even without diagnostic tools, while others reported that patient demand influenced their prescribing decisions. Similar findings have been reported in primary care settings where diagnostic uncertainty, prescriber experience, and patient expectations influence antibiotic use [11,12,16].

Prescribing-related decisions were also influenced by clinical and system-level factors. While laboratory results and patient symptoms were the most frequently reported influences, respondents also identified stock availability, pressure from patients or relatives, limited diagnostic tools, lack of guidelines, and limited supervision. These findings align with studies showing that weak diagnostic capacity, limited guideline access, patient demand, and health-system constraints remain important barriers to effective AMS in LMICs [15,16,21]. Furthermore, a substantial proportion of respondents selected neutral responses when asked about inappropriate prescribing practices. This finding should be interpreted cautiously, as some respondents may have been reluctant to explicitly endorse behaviors perceived as inappropriate. Social desirability bias has been recognized as a potential source of response distortion in health-related surveys [22]. Consequently, the true prevalence of inappropriate or empirical antibiotic prescribing may have been underestimated.

The findings have important implications for PHC AMS. The findings suggest that increased AMR/AMS training, accessible treatment guidelines, improved diagnostic support, and strengthened supervision may improve AMS at the PHC level. These priorities align with Nigeria’s One Health Antimicrobial Resistance National Action Plan 2.0, which emphasizes stewardship strengthening, surveillance, capacity-building, and coordinated AMR control across sectors [23].

This study has several strengths, including its focus on CHWs, a critical but underrepresented group in AMS research, and its assessment of knowledge, training exposure, attitudes, and prescribing-related influences at the PHC level.

This study has some limitations. First, the cross-sectional design limits causal inference, and the reliance on self-reported responses may have introduced recall and social desirability bias. Second, the study did not include objective prescribing data; therefore, reported prescribing practices could not be verified against prescription records. Third, although the findings are relevant to government-owned PHC facilities in Kaduna State, they may not be fully generalizable to private PHC facilities or settings outside Kaduna State. Fourth, the knowledge and attitude score categories were developed by the investigators for descriptive interpretation and were not based on a previously validated scoring instrument, which may limit comparison with studies using different or validated scoring systems. Finally, only descriptive and bivariate analyses were performed; therefore, independent predictors of AMR/AMS knowledge and attitudes could not be identified after adjustment for potential confounding variables.

## Conclusions

This study demonstrates that although CHWs in Kaduna State have high awareness and good knowledge of AMR, prescribing-related decisions may still be influenced by diagnostic uncertainty and contextual pressures. Formal AMS training was strongly associated with improved knowledge and more favorable attitudes. Strengthening training programs, improving access to diagnostic tools, reinforcing guideline use, and enhancing supervision are essential steps toward improving AMS at the PHC level.

## Appendices

Study questionnaire sections used for the present analysis

Sociodemographic and Professional Characteristics

Please tick [✔] the appropriate option(s) where applicable.

1. Age as at last birthday: ______ years

2. Sex: ☐ Male ☐ Female

3. Marital status: ☐ Single ☐ Married ☐ Divorced ☐ Widowed

4. Professional qualification/cadre: ☐ Junior Community Health Extension Worker (JCHEW) ☐ Community Health Extension Worker (CHEW) ☐ Community Health Officer (CHO) ☐ Community Nurse ☐ Others, please specify: _____________

5. Years of professional experience: ☐ Less than 1 year ☐ 1-5 years ☐ 6-10 years ☐ Above 10 years

6. Local Government Area (LGA): __________________

Knowledge and Awareness of Antimicrobial Resistance and Stewardship

Please choose the best answer or tick as instructed.

7. Have you heard of the term “antimicrobial resistance (AMR)”? ☐ Yes ☐ No

8. Antibiotics are effective against which of the following? ☐ Bacteria ☐ Viruses ☐ Both ☐ Don’t know

9. Can inappropriate use of antibiotics cause resistance? ☐ Yes ☐ No ☐ Not sure

10. Completing a full course of antibiotics reduces the risk of AMR. ☐ Yes ☐ No

11. Have you heard of antimicrobial stewardship (AMS)? ☐ Yes ☐ No

12. Antimicrobial stewardship means: ☐ Preventing the sale of antibiotics ☐ Ensuring the appropriate use of antibiotics ☐ Recommending herbal alternatives ☐ Not sure

13. Have you ever received formal training on AMR/AMS? ☐ Yes ☐ No

Attitudes Toward Antimicrobial Prescribing

Please tick the option that best reflects your opinion.

Response options: SA = Strongly agree; A = Agree; N = Neutral; D = Disagree; SD = Strongly disagree.

S/N Statement                                                                                                                   SAANDSD

14. I prescribe antibiotics based on my experience even without diagnostic tools.      ☐☐☐☐☐

15. Patient demand influences my decision to prescribe antibiotics.                              ☐☐☐☐☐

16. I prescribe antibiotics for common cold symptoms.                                                   ☐☐☐☐☐

17.  I prescribe antibiotics for malaria.                                                                                ☐☐☐☐☐

18. I follow a standard treatment guideline when prescribing antimicrobials.                ☐☐☐☐☐

19. I document all antimicrobial prescriptions.                                                                  ☐☐☐☐☐

Factors Influencing Antimicrobial Prescription

20. What factors influence your decision to prescribe antibiotics? Select all that apply.

☐ Patient symptoms ☐ Laboratory results ☐ Pressure from patients and relatives ☐ Stock availability in the facility ☐ Previous patient response to similar symptoms ☐ Lack of supervision or oversight ☐ Lack of diagnostic tools ☐ Lack of guidelines

21. How often do you receive supervision on prescribing practices? ☐ Never ☐ Rarely ☐ Occasionally ☐ Regularly ☐ Always
